# Long non-coding RNA SPRY4-IT1 promotes cell proliferation and invasion by regulation of Cdc20 in pancreatic cancer cells

**DOI:** 10.1371/journal.pone.0193483

**Published:** 2018-02-28

**Authors:** Wenhao Guo, Kunhong Zhong, Heng Wei, Chunlai Nie, Zhu Yuan

**Affiliations:** 1 Department of Abdominal Oncology, Cancer Center and State Key Laboratory of Biotherapy, West China Hospital, West China Medical School, Sichuan University, Chengdu, Sichuan Province, People’s Republic of China; 2 Lab of Biotherapy and Cancer Center, West China Hospital, Sichuan University, Chengdu, People’s Republic of China; Universitat des Saarlandes, GERMANY

## Abstract

Accumulating evidence has demonstrated that long non-coding RNAs (lncRNAs) play a critical role in the development of human cancers including pancreatic cancer. Long non-coding RNA SPRY4-IT1 (sprouty4-intron transcript 1) has been reported to play an oncogenic role in various types of human carcinomas. However, the role of SPRY4-IT1 in pancreatic cancer is unclear. The objective of this study was to determine the function of SPRY4-IT1 on proliferation and invasion in pancreatic cancer. In the current study, we dissected the function and mechanism of SPRY4-IT1 by multiple approaches including Real-time RT-PCR, Western blotting analysis, MTT assay, Wound healing assay, Transwell assay, and transfection. We found that down-regulation of SPRY4-IT1 inhibited cell growth and induced cell apoptosis in pancreatic cancer cells. Moreover, SPRY4-IT1 knockdown induced cell cycle arrest at G0/G1 phase. Furthermore, inhibition of SPRY4-IT1 retarded cell migration and invasion in pancreatic cancer cells. Overexpression of SPRY4-IT1 enhanced cell growth and invasion, and inhibited cell apoptosis in pancreatic cancer cells. Mechanistically, suppression of SPRY4-IT1 inhibited the expression of Cdc20 in pancreatic cancer cells. Our findings demonstrated that inhibition of SPRY4-IT1 could be a potential therapeutic approach for the treatment of pancreatic cancer.

## Introduction

Pancreatic cancer is one of the highly aggressive tumors in human [1]. The expected numbers of new pancreatic cancer cases and deaths in 2017 in the United States are 53,670 and 43,090, respectively [2]. The five-year relative survival rate is currently 8% in the United States. This low rate is partly because more than one-half of pancreatic cancer patients are diagnosed at a distant stage [2]. Although several treatment strategies including surgery of tumor resection, chemotherapy, and immunotherapy have been used, the outcomes of pancreatic cancer patients are still bad [3, 4]. Thus, it is highly urgent to explore the molecular mechanism of pancreatic cancer progression and to find the new therapeutic targets for the treatment of pancreatic cancer.

Emerging evidence has revealed that long non-coding RNAs (lncRNAs), a subgroup of noncoding RNAs, play a critical role in the development of human cancers including pancreatic cancer [5]. It has been known that lncRNAs are longer than 200 nucleotides, but have little or no function of protein-coding capacity [6]. Recent studies have demonstrated that lncRNAs govern gene expression via chromosome remodeling, transcription and post-transcriptional processes. Therefore, lncRNAs could regulate multiple cellular precession including proliferation, apoptosis, cell cycle, migration, and invasion [7]. Without a doubt, abnormal expression of lncRNAs could contribute to tumor development and progression [8]. In line with this, lncRNAs have been reported to play pivotal roles in various types of human carcinomas including SPRY4-IT1 [8, 9]. It has been documented that SPRY4-IT1 is transcribed from the second intron of the SPRY4 gene [9]. Accumulating evidence has suggested that SPRY4-IT1 plays an oncogenic role in human cancers [9]. However, the role of SPRY4-IT1 in pancreatic cancer is unclear. In this study, we determined the function of SPRY4-IT1 in the regulation of proliferation, apoptosis, cell cycle, migration and invasion in pancreatic cancer. We further explored the potential mechanism of SPRY4-IT1-mediated tumor progression. Our findings suggest that inhibition of SPRY4-IT1 could be a potential therapeutic approach for the treatment of pancreatic cancer.

## Results

### Down-regulation of LncRNA SPRY4-IT1 inhibited cell growth

**Fig 1:**
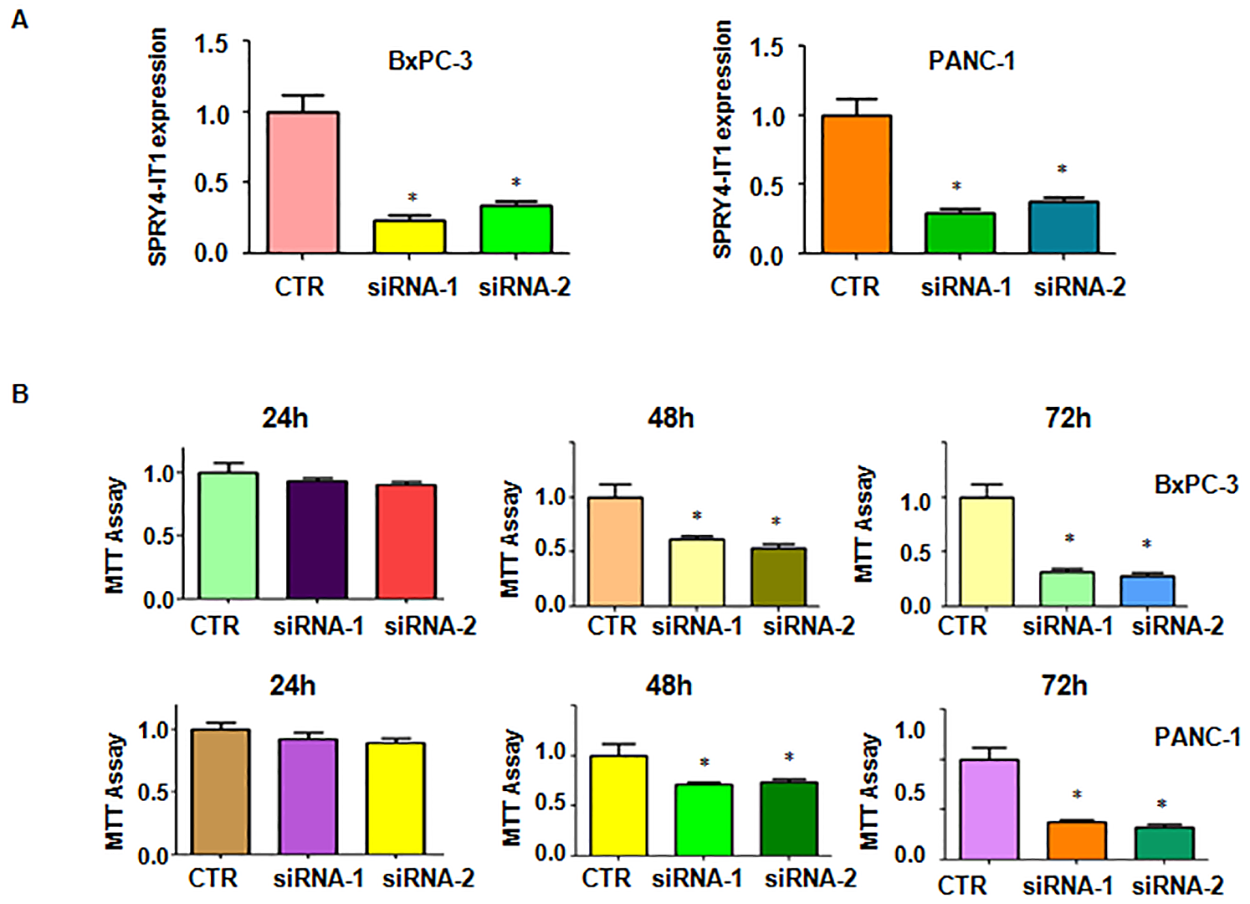
Effect of SPRY4-IT1 depletion on cell growth. (A) Real-time RT-PCR was performed to measure SPRY4-IT1 expression in pancreatic cancer cells after SPRY4-IT1 siRNA transfection. (B) MTT assay was conducted to detect cell proliferation in pancreatic cancer cells after SPRY4-IT1 siRNA transfection for 24 h, 48 h, and 72 h, respectively.

To explore the function of SPRY4-IT1 in pancreatic cancer cells, BxPC-3 and PANC-1 cells were transfected with SPRY4-IT1 siRNA to down-regulate the expression of SPRY4-IT1. The efficacy of SPRY4-IT1 siRNA transfection was validated by real-time RT-PCR. Our results showed that SPRY4-IT1 siRNA significantly reduced the SPRY4-IT1 expression in both pancreatic cancer cell lines (Fig 1A). To determine whether SPRY4-IT1 plays a role on cell growth, we conducted MTT assay in pancreatic cancer cells after SPRY4-IT1 siRNA transfectionn. We found that down-regulation of SPRY4-IT1 inhibited cell growth in both BxPC-3 and PANC-1 cells (Fig 1B). Our results further demonstrated that SPRY4-IT1 siRNA 1 exhibited cell growth inhibition at greater degree. Therefore, we used SPRY4-IT1 siRNA 1 for our following further studies.

### Down-regulation of LncRNA SPRY4-IT1 induced cell apoptotic death

**Fig 2:**
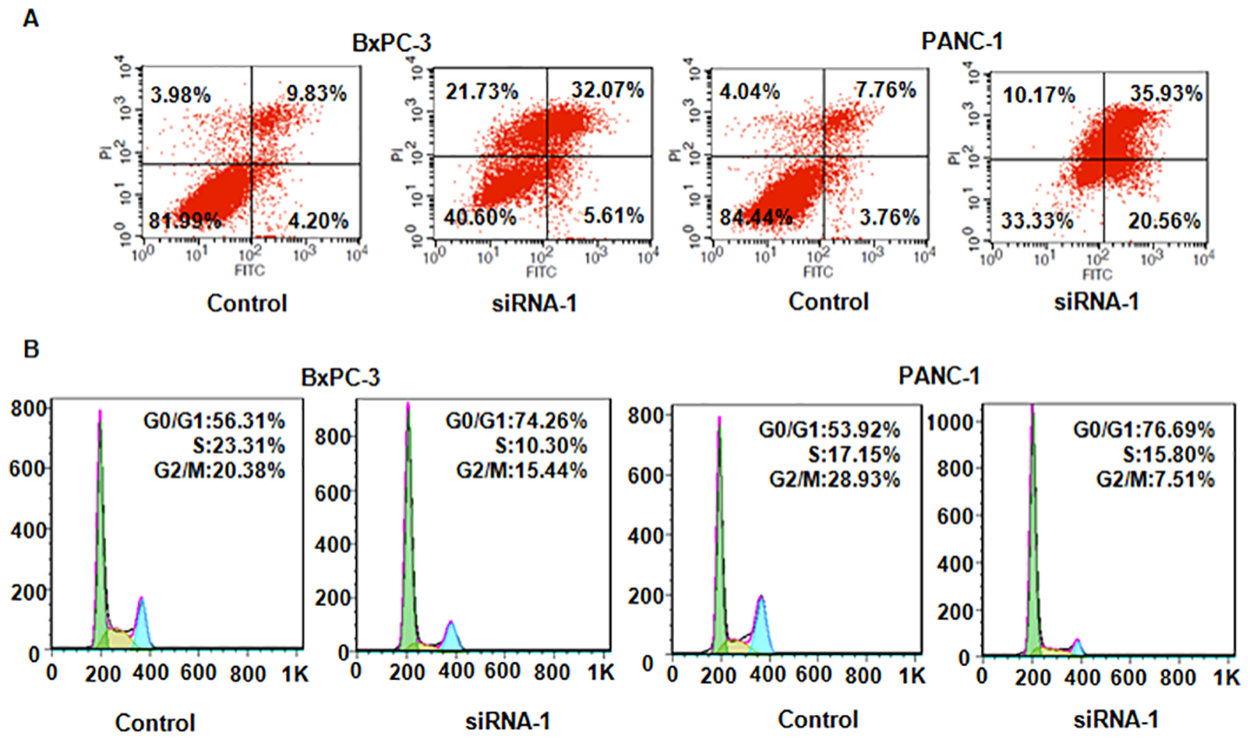
Effect of SPRY4-IT1 depletion on apoptosis, and cell cycle arrest. (A) Apoptotic cell death was measured using Annexin V-FITC/PI method in pancreatic cancer cells after SPRY4-IT1-1 siRNA transfection for 48 hours. Control: control siRNA; siRNA-1: SPRY1-IT1 siRNA-1. (B) Cell cycle analysis was performed in pancreatic cancer cells after SPRY4-IT1 siRNA-1 transfection for 72 hours.

To further determine whether SPRY4-IT1 could induce cell apoptosis, Annexin V-FITC/PI and FACS were used to measure the percentage of cell apoptotic death in pancreatic cancer cells after SPRY4-IT1 siRNA transfection. We observed that the percentage of apoptotic cells was reduced in BxPC-3 and PANC-1 cells transfected with SPRY4-IT1 siRNA (Fig 2A). This result suggested that down-regulation of SPRY4-IT1 induced cell apoptosis in pancreatic cancer cells.

### Down-regulation of LncRNA SPRY4-IT1 induced cell cycle arrest

To further define how SPRY4-IT1 regulated cell growth, PI staining and flow cytometry were used to measure the cell cycle phases in pancreatic cancer cells after SPRY4-IT1 siRNA transfection. Our data showed that the percentage of G0/G1 phase was increased from 56.31% to 74.26% in BxPC-3 cells after SPRY4-IT1 siRNA transfection (Fig 2B). Similarly, the proportion of G0/G1 phase was increased from 53.92% in control group to 76.69% in SPRY4-IT1 siRNA transfected PANC-1 cells (Fig 2B). These data revealed that down-regulation of SPRY4-IT1 induced cell cycle arrest into G0/G1 phase in pancreatic cancer cells.

### Down-regulation of LncRNA SPRY4-IT1 inhibited cell migration and invasion

**Fig 3:**
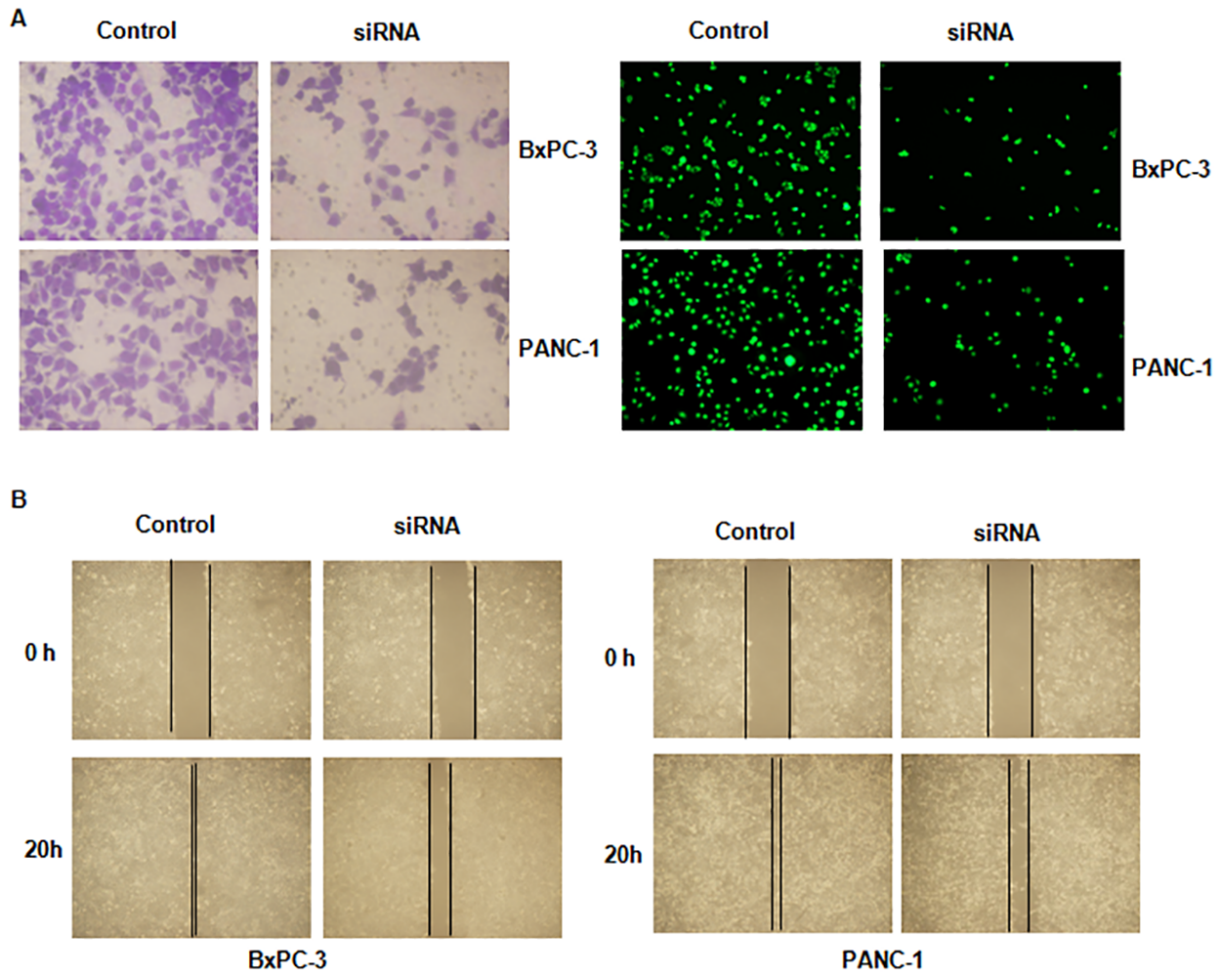
Effect of SPRY4-IT1 depletion on cell migration and invasion. (A) Cell invasion was measured using Transwell inserts with Matrigel in pancreatic cancer cells after SPRY4-IT1 siRNA transfection for 24 hours. Invasive cells were stained with Giemsa solution (Left panel) and calcein AM (Right panel). (B) Cell migration was detected using Wound-healing assay in pancreatic cancer cells after SPRY4-IT1 siRNA transfection for 20 hours.

To determine whether SPRY4-IT1 could regulate cell migration in pancreatic cancer cells, a scratch wound-healing assay was performed in BxPC-3 and PANC-1 cells transfected with SPRY4-IT1 siRNA. We observed that down-regulation of SPRY4-IT1 inhibited cell migration in both pancreatic cancer cell lines (Fig 3A). To further validate the function of SPRY4-IT1 on cell invasion, we measured cell invasive activity using Transwell inserts with Matrigel. Our results from invasion assay showed that SPRY4-IT1 siRNA treatment suppressed cell invasion in pancreatic cancer cells (Fig 3B). Altogether, suppression of SPRY4-IT1 inhibited cell motility activity.

### LncRNA SPRY4-IT1 promoted cell growth and invasion

**Fig 4:**
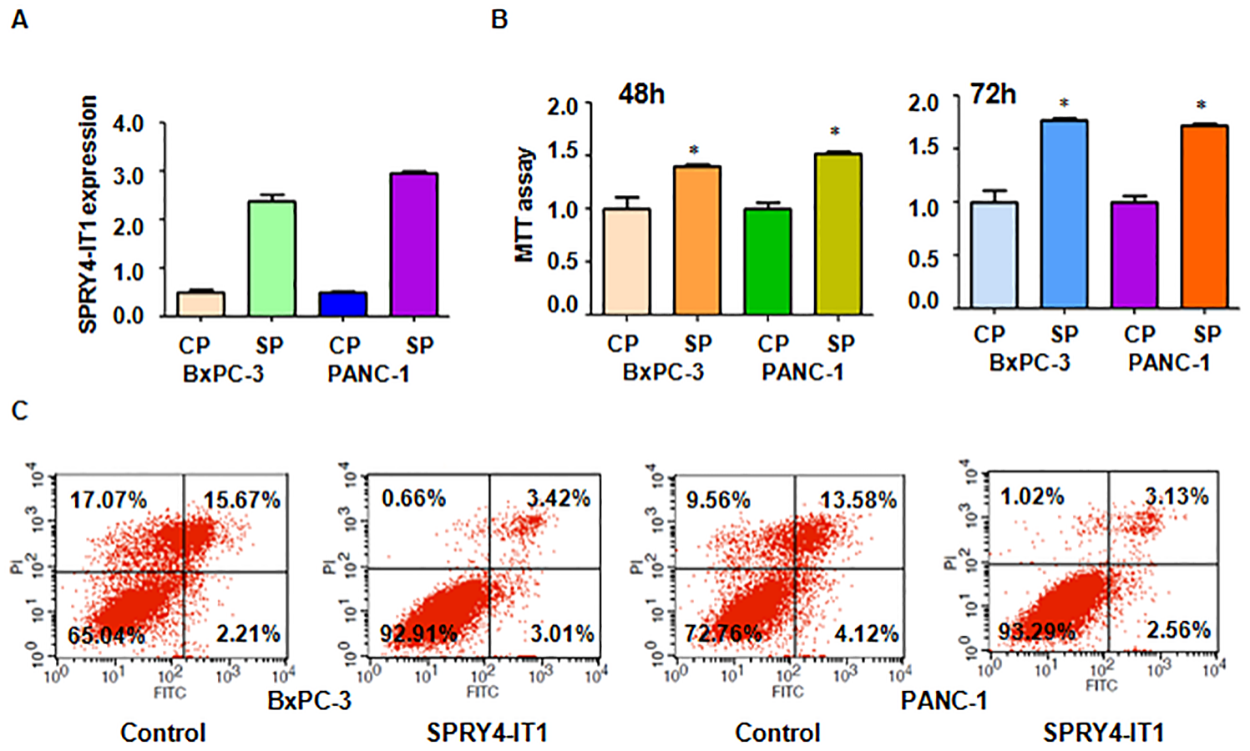
Effect of SPRY4-IT1 overexpression on cell growth and apoptosis. (A) Real-time RT-PCR was performed to measure SPRY4-IT1 expression in pancreatic cells after plasmid with SPRY4-IT1 cDNA transfection. CP: Control plasmid; SP: SPRY4-IT1 plasmid. (B) MTT assay was conducted to detect cell proliferation in pancreatic cancer cells after plasmid including SPRY4-IT1 cDNA transfection for 48 h and 72h. (C) Apoptotic cell death was measured using Annexin V-FITC/PI method in pancreatic cancer cells after plasmid containing SPRY4-IT1 cDNA transfection for 48 hours.

**Fig 5:**
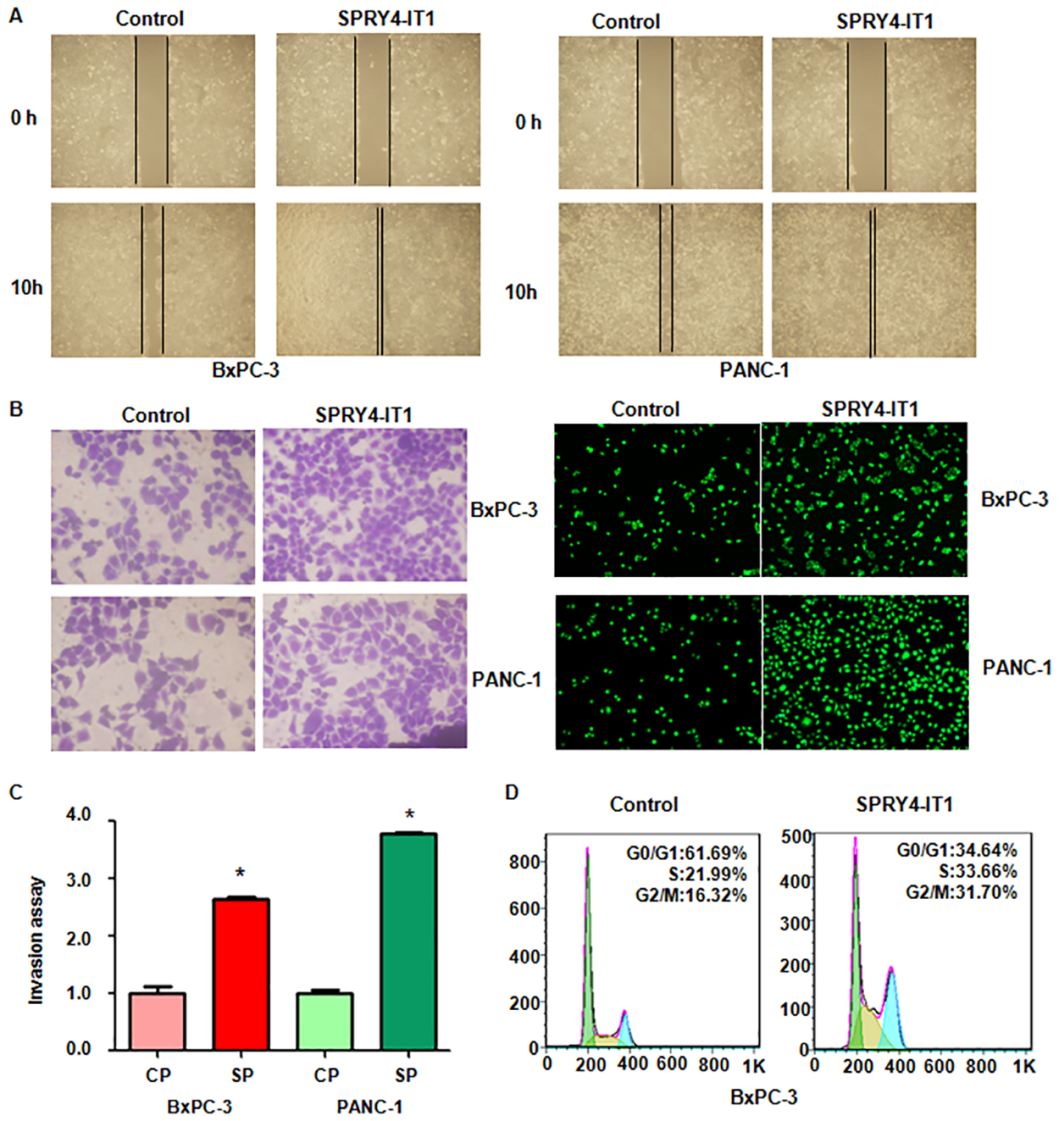
Effect of SPRY4-IT1 up-regulation on cell migration and invasion. (A) Cell migration was detected using Wound-healing assay in pancreatic cancer cells after SPRY4-IT1 cDNA transfection for 10 hours. (B) Cell invasion was measured using Transwell inserts with Matrigel in pancreatic cancer cells after SPRY4-IT1 cDNA transfection for 24 hours. Invasive cells were stained with Giemsa solution (Left panel) and calcein AM (Right panel). (C) Quantitative results are illustrated for panel B at right section. CP: Control plasmid; SP: SPRY4-IT1 plasmid. (D) Cell cycle analysis was performed in pancreatic cancer cells after SPRY4-IT1 cDNA transfection for 72 hours.

To further confirm the function of SPRY4-IT1 in pancreatic cancer cells, we overexpressed SPRY4-IT1 in BxPC-3 and PANC-1 cells using recombinant lentiviruses containing full length SPRY4-IT1 (Fig 4A). We found that overexpression of SPRY4-IT1 promoted cell growth in both pancreatic cancer cell lines (Fig 4A). Moreover, overexpression of SPRY4-IT1 inhibited cell apoptosis in pancreatic cancer cells (Fig 4B). Furthermore, up-regulation of SPRY4-IT1 promoted cell migration and invasion in both pancreatic cancer cell lines (Fig 5B and 5C). Overexpression of SPRY4-IT1 enhanced cell cycle progression (Fig 5D). Taken together, SPRY4-IT1 plays an oncogenic role in pancreatic cancer cells.

### SPRY4-IT1 regulated Cdc20 expression

**Fig 6:**
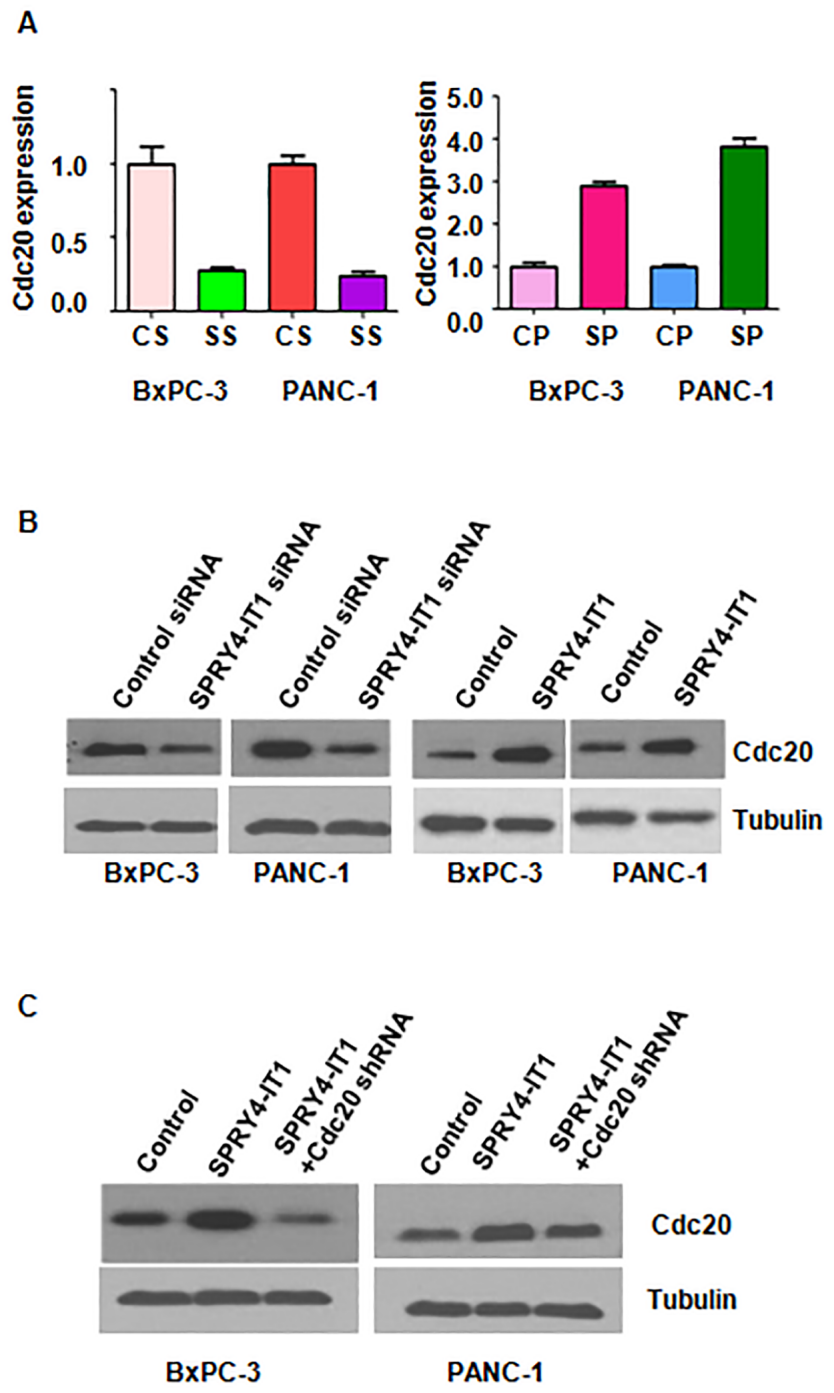
Effect of SPRY4-IT1 on the expression of Cdc20. (A) The Cdc20 mRNA level was measured by real-time RT-PCR in pancreatic cancer cells after SPRY4-IT1 siRNA or cDNA transfection. CS: Control siRNA; SS: SPRY4-IT1 siRNA. CP: Control plasmid; SP: SPRY4-IT1 plasmid. (B) The Cdc20 protein level was measured by Western blotting analysis in cells after SPRY4-IT1 siRNA or SPRY4-IT1 cDNA transfection. (C) The Cdc20 expression was measured by Western blotting analysis in cells after SPRY4-IT1 cDNA transfection plus Cdc20 shRNA infection.

To reveal the potential molecular mechanism of SPRY4-IT1 in pancreatic cancer cells, we measured the expression of Cdc20 (cell division cycle 20) using Western blotting analysis. We found that downregulation of SPRY4-IT1 inhibited the expression of Cdc20 in BxPC-3 and PANC-1 cells (Fig 6A and 6B, S1 Fig). In line with this, overexpression of SPRY4-IT1 increased Cdc20 expression in pancreatic cancer cells (Fig 6A and 6B, S1 Fig).

### Depletion of Cdc20 decreased cell growth and invasion caused by SPRY4-IT1

**Fig 7:**
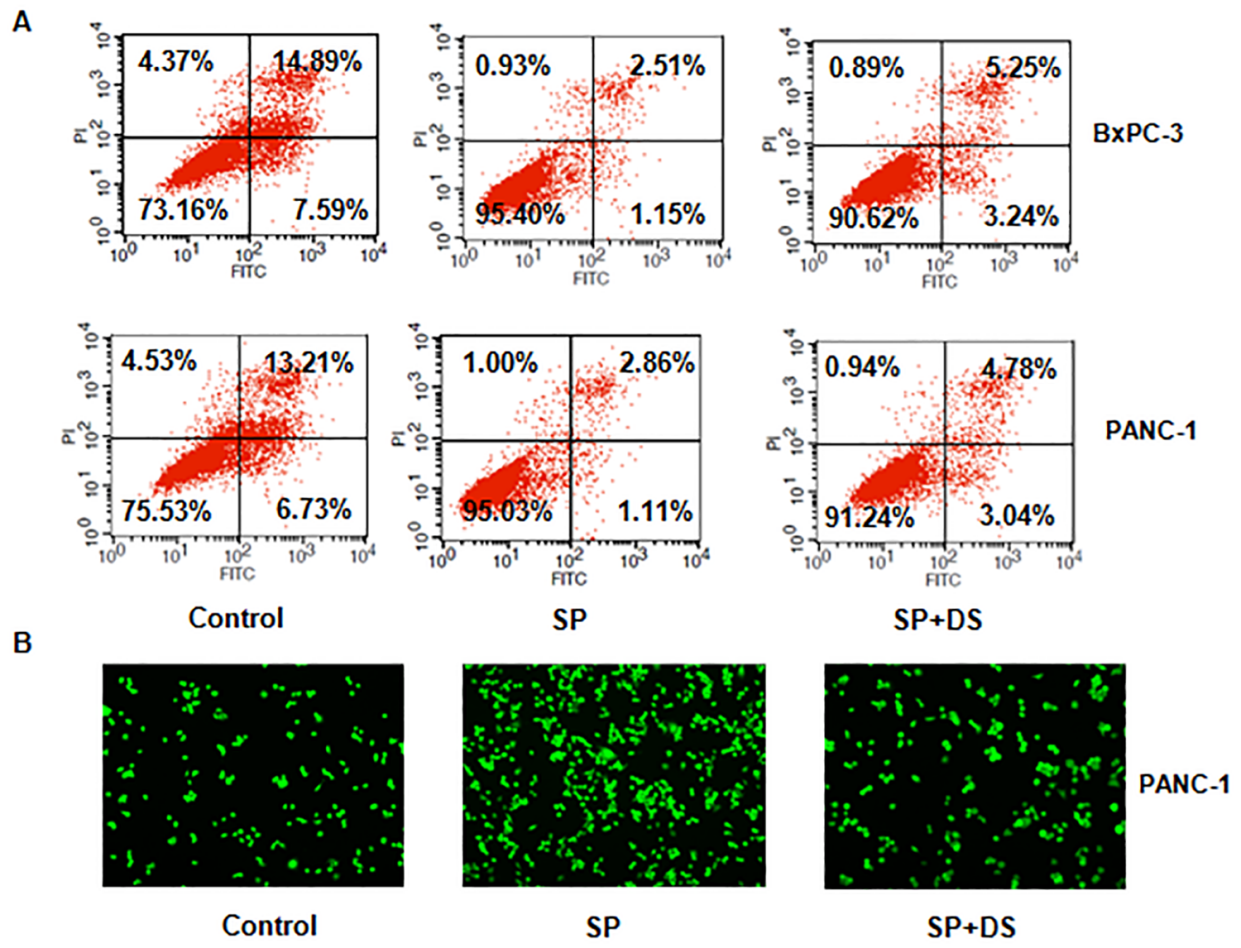
Effect of SPRY4-IT1 on the cell apoptosis and invasion. (A) Apoptotic cell death was measured using Annexin V-FITC/PI method in cells after SPRY4-IT1 cDNA transfection plus Cdc20 shRNA infection for 48 hours. SP: SPRY4-IT1 plasmid; SP+DS: SPRY4-IT1 plus Cdc20 shRNA. (B) Cell invasion was measured using Transwell inserts with Matrigel in cells after SPRY4-IT1 cDNA transfection plus Cdc20 shRNA infection for 24 hours. SP: SPRY4-IT1 plasmid; SP+DS: SPRY4-IT1 plus Cdc20 shRNA.

Next, to further investigate whether Cdc20 was involved in SPRY4-IT1-mediated tumor progression, we performed a rescue assay. SPRY4-IT1 and Cdc20 shRNA were simultaneously transfected into BxPC-3 cells. We observed that Cdc20 expression is reduced in SPRY4-IT1-transfected cells after Cdc20 shRNA infection (Fig 6C and S1 Fig). Our MTT assay results showed that depletion of Cdc20 decreased cell growth caused by SPRY4-IT1 upregulation (Fig 7A). Furthermore, Cdc20 down-regulation enhanced cell apoptosis in SPRY4-IT1-transfected cells (Fig 7B). Moreover, Transwell chambers assay results demonstrated that silencing of Cdc20 inhibited cell invasion induced by SPRY4-IT1 upregulation (Fig 7B). These finding suggested that SPRY4-IT1 could promote tumor progression via regulation of Cdc20 in pancreatic cancer cells.

## Discussion

In the present study, we investigated the biological function of SPRY4-IT1 on cell proliferation, cell cycle, apoptosis, and motility activity in pancreatic cancer cells. We found that down-regulation of SPRY4-IT1 inhibited cell growth and induced cell cycle arrest at G0/G1 phase as well as cell apoptosis in pancreatic cancer cells. Consistently, upregulation of SPRY4-IT1 promoted cell growth and inhibited apoptosis. Moreover, SPRY4-IT1 inhibited cell migration and invasion, while overexpression of SPRY4-IT1 enhanced cell motility activity. Mechanistically, suppression of SPRY4-IT1 inhibited the expression of Cdc20, whereas overexpression of SPRY4-IT1 increased Cdc20 level in pancreatic cancer cells. Our findings indicated that SPRY4-IT1 exerts its function in part through regulation of Cdc20 in pancreatic cancer.

A line of evidence suggests that SPRY4-IT1 was critically involved in tumorigenesis [10]. For example, some studies have shown that SPRY4-IT1 was up-regulated in a variety of human cancers including esophageal squamous cell carcinoma (ESCC) [11], renal cancer [12], breast cancer [13], gastric cancer [14], bladder cancer [15], and HCC (hepatocellular carcinoma) [16]. More importantly, SPRY4-IT1 overexpression was associated with poor prognosis in various types of human malignancies such as renal cancer [12], ESCC [11], gastric cancer [14], bladder cancer [15], HCC [16], glioma [17], cervical cancer [18], colorectal cancer [19], ovarian cancer [20], and lung cancer [21]. For instance, increased SPRY4-IT1 expression was reported to be associated with a larger tumor size and an advanced pathological stage in breast cancer patients [13]. SPRY4-IT1 levels were highly positively correlated with tumor size, TNM stage, invasion depth, distant metastasis, and reduced OS (overall survival) and DFS (disease-free survival) in gastric cancer [14]. Recently, Zhou et al reported that SPRY4-IT was concerned with the poor prognosis and contributed to the progression of thyroid cancer [22]. These reports suggest that SPRY4-IT1 plays an oncogenic role in human cancers.

Recent studies have revealed that knockdown of SPRY4-IT1 led to defects in cell growth, differentiation, and higher rates of apoptosis in melanoma cell lines [23]. Similarly, SPRY4-IT1 down-regulation reduced cell proliferation in vitro and in vivo in ESCC cells [11]. In line with this, depletion of SPRY4-IT1 reduced renal cancer cell proliferation, migration and invasion [12]. The elevated expression of SPRY4-IT1 increased rate of wound closure in melanoma cells [23]. Knockdown of SPRY4-IT1 reduced cell invasiveness and migration in ESCC cells [11]. In the present study, for the first time, we reported that SPRY4-IT1 enhanced cell proliferation, migration and invasion in pancreatic cancer cells. Therefore, down-regulation of SPRY4-IT1 could be useful for treating patients with pancreatic cancer.

Multiple studies have unraveled that SPRY4-IT1 regulated its downstream targets to exert its function. Sun et al reported that EZH2 (enhancer of zeste homolog 2)-mediated epigenetic suppression of SPRY4-IT1 promoted cell proliferation and metastasis through regulation of EMT (epithelial-mesenchymal transition) in NSCLC (non-small cell lung cancer) cells [24]. Interestingly, SPRY4-IT1 promoted tumor cell proliferation and invasion through activation of EZH2 in HCC [25]. Overexpression of SPRY4-IT1 promoted EMT via the regulation of E-cadherin and vimentin expression in NSCLC cells [24], ESCC cells [26, 27], colorectal cancer [18, 28], and ovarian cancer cells [29]. In keeping with this report, knockdown of SPRY4-IT1 suppressed glioma cell proliferation, metastasis and EMT [30]. Moreover, SPRY4-IT1 promoted EMT through association with Snail1 in osteosarcoma [31]. SPRY4-IT1 knockdown induced apoptosis via lipin2-mediated alterations in lipid metabolism in melanoma cells [32]. SPRY4-IT1 increased the proliferation through upregulation of ZNF703 expression in human breast cancer cells [13]. SPRY4-IT1 promoted cell proliferation, migration and invasion partially through regulation of certain cyclins and MMPs (matrix metalloproteinases)-related genes in gastric cancer [14]. Yu et al found that SPRY4-IT1 promoted development of hepatic cellular carcinoma via interacting with ERRα and predicted poor prognosis [33]. Recent study showed that SPRY4-IT1 sponged miR-101-3p to promote proliferation and metastasis through upregulation of EZH2 in bladder cancer cells and colorectal cancer cells [34, 35]. In the current study, we identified that SPRY4-IT1 could upregulate the expression of Cdc20 oncoprotein in pancreatic cancer. Higher expression of Cdc20 has been observed in a variety of human cancers and is correlated with poor prognosis [36]. Therefore, SPRY4-IT1 could exert its oncogenic function in part via upregulation of Cdc20. Without a doubt, further in-depth explore whether SPRY4-IT1 is a valuable target for therapeutic intervention in pancreatic cancer is required.

## Materials and methods

### Cell culture and reagents

BxPC-3 (K-ras wild-type, p53 and p16 mutants, Rb positive) and PANC-1 (K-ras mutant, p53 and p16 mutants, Rb positive) cells were purchased from the American Type Culture Collection (Manassas, VA, USA). Cells were maintained in DMEM containing 10% fetal bovine serum at 37°C in 5% CO_2_ humidified incubator. MTT [3-(4,5-dimethythiazol- 2-yl)-2,5-diphenyl tetrazolium bromide] are obtained from Sigma (St. Louis, MO, USA). Anti-Cdc20, anti-tubulin, and the secondary antibodies were purchased from Cell Signaling Technology Company.

### Cell tranfection

BxPC-3 and PANC-1 cells were seeded in six-well plates and transfected with lncRNA SPRY4-IT1 siRNA or the scrambled siRNA as the negative control using lipofectamine 2000 according to manufacturer’ s instructions [37]. The siRNA negative control: GGC TAC GTC CAG GAG CGC A, SPRY4-IT1 siRNA 1: CCC AGA ATG TTG ACA GCT GCC TCT T, SPRY4-IT1 siRNA 2: TGG AGG GTT ATG GGA GCC TGT GAA T. The recombinant lentiviruses containing full length SPRY4-IT1 and the nonspecific control were obtained from GenePharma Company (Shanghai, China). The alterations of SPRY4-IT1 in pancreatic cancer cells were validated by real-time PCR.

### Quantitative reverse transcription-PCR

Total RNA in transfected pancreatic cancer cells was extracted using TRIzol reagent (Invitrogen, Carlsbad, CA) according to manufacturer’ s instructions. Then, RNA was reverse-transcribed into complementary DNA by the Reverse cDNA synthesis kit. Real-time PCR was conducted on the ABI 7500 cycler using SYBR Green PCR kit. The primer sequences were as following: SPRY4-IT1: forward 5’-AGC CAC ATA AAT TCA GCA GA-3’, reverse 5’-GAT GTA GGA TTC CTT TCA-3’. Cdc20, forward primer 5′- GAC CAC TCC TAG CAA ACC TGG -3′, reverse primer 5′-GGG CGT CTG GCT GTT TTC A-3′.

### MTT assay

The transfected cells (5 ×10^3^) were seeded in 96-well culture plates. At the indicated time points, the cells were treated with MTT by adding it to the medium. Cells were incubated at 37°C for 4 hours and then the medium was removed. DMSO was added for 30 minutes to lyse the cells. Optical density (OD) was measured at 490 nm using a micro-plate reader [38].

### Cell apoptosis assay

The transfected pancreatic cancer cells were cultured in 6-well plates for 48 hours. Then, cells were harvested and resuspended in 500μl binding buffer with 5μl Propidium iodide (PI) and 5μ FITC-conjugated anti-Annexin V antibody. Apoptosis was measured using a FACScalibur flow cytometer (BD, USA) as described before [38].

### Cell cycle analysis

The transfected pancreatic cancer cells were seeded in a six-well plate for overnight. After 48 hours, cells were harvested, washed and resuspended in 70% cold alcohol and kept at 4°C overnight. Then, cells were suspended in 1 × 10^6^ cells/ml in PBS. Subsequently, cells were incubated with 0.1mg/ml RNase I and 50 mg/ml PI at 37°C for 30 min. Cell cycle was further detected with a FACScalibur flow cytometer (BD, USA).

### Wound healing assay

The transfected cells were seeded in 6-well plates for 48 hours. After the cells get 100% gather, the cell momolayer in each well was scraped using a 200 μl pipette tip to generate a wound. Photographic images were taken by using microscope from each well at 0 h, 10 h or 20 h post-injury time points.

### Invasion assay

The invasion assay was conducted in transfected pancreatic cancer cells by 24-well Transwell inserts with Matrigel coating. Specifically, cells were seeded into the upper chamber of an 8μm pore size insert. The cells were allowed to invade the bottom chamber containing 10% FBS. After incubation for 24h, the invasive cells attached to the lower membrane surface were fixed with 4% paraformaldehyde and stained with Giemsa solution and 4 μg/mL calcein AM, respectively. The stained invasive cells were photographed.

### Western blotting assay

To determine the expression of Cdc20 in transfected pancreatic cancer cells, we conducted Western blotting analysis. The cells were lysed in the cold lysis buffer in the presence aprotinin, leupeptin, PMSF, and phosphatase inhibitor cocktails II and III (Sigma) The protein extracts were separated on SDS-PAGE and transferred to PVDF membrane. The membrane was incubated with indicated antibodies and detected using an enhanced chemiluminescence kit as described previously [37]. The anti-Cdc20 (1:1000), anti-tubulin (1:2500), and the secondary antibodies (1:3000) were used in this assay.

### Statistical analysis

All the statistical analyses were performed using GraphPad Prism 4.0 (Graph pad Software, La Jolla, CA) by Student’s test. All the data were expressed as mean ± SD (standard deviation). Moreover, p<0.05 was considered statistically significant.

## Supporting information

S1 Fig Original images for Fig 6B and 6C. (DOCX)
