# Supplementary material for: Long non-coding RNA SPRY4-IT1 promotes cell proliferation and invasion by regulation of Cdc20 in pancreatic cancer cells
Source: PLoS One. 2018 Feb 28;13(2):e0193483. doi: 10.1371/journal.pone.0193483 (PMC5831108; doi:10.1371/journal.pone.0193483)

Figure 6B

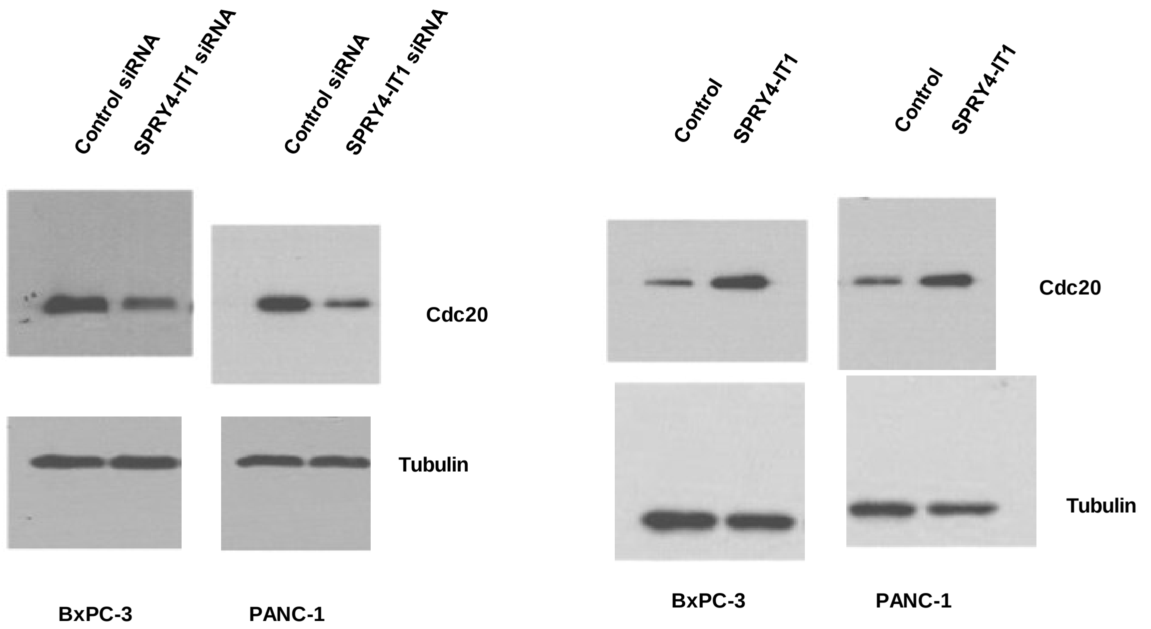

Figure 6C

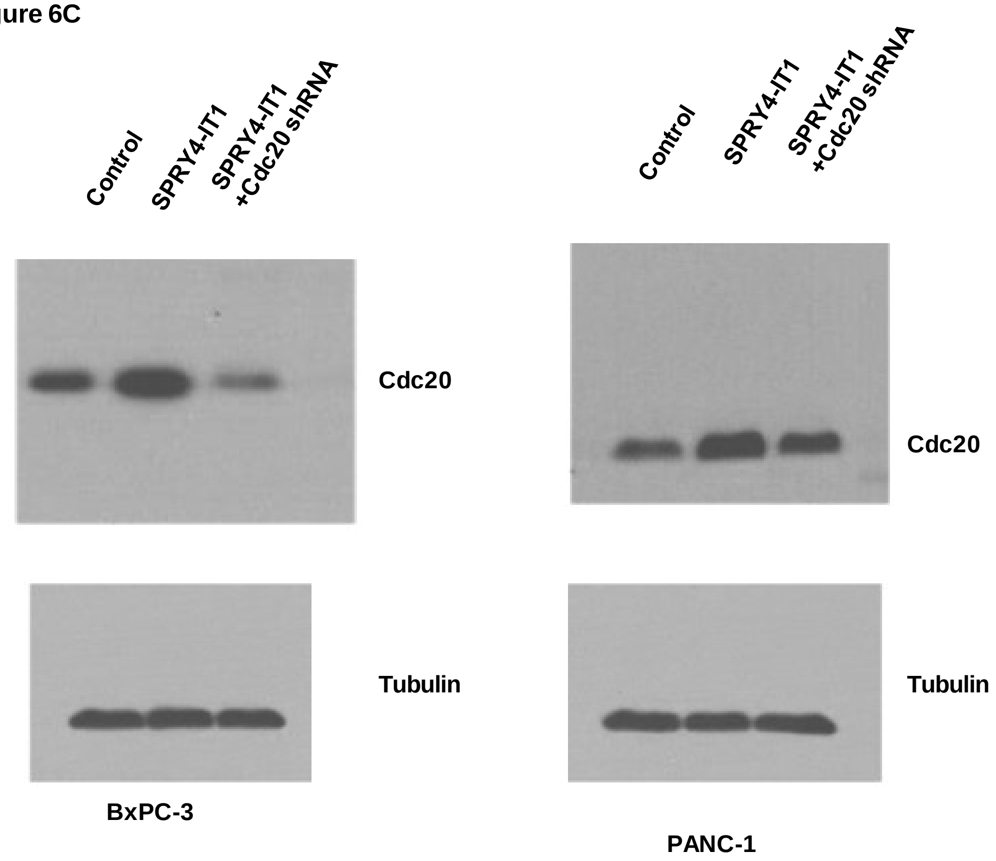

Supplement: Supplementary file 1 [file pone.0193483.s001.docx]
